# The London Hospital

**Published:** 1914-06-13

**Authors:** 


### THE DEPARTMENT OF MODERN NURSING.*

### III.—The London Hospital.

THE London Hospital, with 922 beds and cots, is the largest charitable institution in England for the care of the sick poor. It was founded in the year 1740, and has been seven times enlarged; the last occasion, in 1904, involved the reconstruction and modernising of the entire establishment. During 1912 a total of 16,827 in-patients were nursed in the wards, or a daily average of 817. Of these in-patients 9,005 were operated on. There were 220,729 out-patients during the year, and in this department 12,550 operations of various kinds took place. It is evident that unrivalled opportunities for learning the duties of a nurse are afforded in such an establishment.

### CASTING THE NET.

The full nursing staff in 1912 numbered 444, in addition to a private nursing staff of 267. About 400 of these are passing through the hospital in successive stages in fulfilment of their four years' term of agreement. The number of nurses who gained certificates at the expiry of their two years' term of training last year was 116. The result of this onward movement on the part of the staff is a large and ceaseless demand for new probationers to fill the vacancies made by promotions. To satisfy the claims of the hospital and private nursing staff over 200 new probationers are needed every year. It is one of the heavy responsibilities of the Matron to find this number of *suitable* candidates, to separate the wheat from the chaff, and select thirty women, sound in mind and body, well fitted for hospital work, from the miscellaneous crowd of applicants every seven weeks. From all parts of the United Kingdom, from many places in the Colonies, a shower of applications pour into the Matron's department, to be sorted into two groups, those who are obviously unfit and those likely to prove good candidates. About a dozen of the latter, who comply with age and other regulations, are interviewed every week. The previous education and occupation of the candidate, her health and disposition, her appearance and manner are passed under review. And a momentous decision has to be made for or against. The instinct for pronouncing on character swiftly and detecting qualities under an unpromising manner develops by experience until it is rarely at fault.

### THE PRELIMINARY TRAINING SCHOOL.

It is obvious that the process of admitting to the wards a group of thirty young women every two months or so who have had no previous experience of hospital work must have constituted a tremendous strain on the staff right through the establishment. This is now obviated by the admirably organised preliminary training school at Tredegar House, of which plans and a description were published in the pages of THE HOSPITAL (July 20, 1912, p. 413). The building, which is situated in Bow Road, affords accommodation for thirty pupils. The course lasts seven weeks, and, reckoning for a necessary holiday in August, it is calculated that the school can be filled six and a half times in the course of the year and turn out close upon 200 pupils. The practical instruction given includes the elements of housework, practised by the pupils on the rooms in which they live. They are taught to be scrupulously neat and orderly in the use of household implements and in their own persons, to be alert and punctual. In practical nursing they learn all such details as can be taught by way of preparation for ward duties, including the care of a dummy patient in a specimen ward, fitted up with minute attention to detail. Lessons on bandaging and on the subjects which make part of a first-aid course and on sick-room cookery, together with lectures and classes on elementary anatomy, physiology, and hygiene, are provided, and ability to learn is tested by written and *viva voce* examinations. Weekly records of each pupil's progress are received by the Matron of the London Hospital, so that at the expiry of this training all are well known, and can be passed on to their trial month in the wards without much misgiving. The plausible, neurotic women who, alas! so often desire to take up nursing, not to mention the other unsuitable people, are almost infallibly weeded out after a few weeks of searching preparation under the experienced and sympathetic Sister-in-Charge at Tredegar House.

No payment is required of the pupils, experience tending to show that this would exclude altogether a very large proportion of those who subsequently turn into good nurses. Room is found, however, for one or two girls desiring an insight into nursing, and these pay £15 15s. for the course. The training costs the hospital £1,200 a year, but as it ensures that new probationers are of use from the moment of entering the wards it may be considered a valuable, even an indispensable, adjunct to the training school. It does not quite suffice for the needs of the hospital, and every week one or two probationers are admitted straight to the wards. These receive in the course of their training special lessons to compensate for the lack of the preliminary preparation.

### A TWO-YEARS' COURSE.

The term of training lasts two years, at the end of which time probationers are eligible to receive the London Hospital certificate of training. Every probationer, however, enters upon an agreement to stay four years at the hospital and to do such work as she is directed to do during this period.

In the year 1912 the training school contained 117 second-year probationers, 109 first-year probationers, 20 paying probationers in their first and second years, and 20 candidates on trial.

* Articles I. and II., on St. Bartholomew's Hospital and St. George's Hospital respectively, appeared on May 16 (p. 189) and May 30 (p. 247).

### The First Eighteen Months.

We must now follow the group of pupil-probationers from Tredegar House to their place in the London Hospital. The first month is " on trial." The general system in regard to the probationers is to change them from one ward to another all over the hospital once a month. They are sent for one month to a medical ward and are then changed to a surgical, or vice versa. They pass from male wards to female in the same rotation, so far as can be arranged, remaining on occasionally an extra fortnight, if the Sister of the ward believes that it will be for their advantage. Practical instruction is going on the whole time, the Sister of each ward gathering the probationers about her for informal classes on every detail of ward work. Constant vigilance is used by the Assistant Matrons to ensure that this branch of training is not neglected in any ward. Three courses of lectures are delivered every year, and all probationers attend these until they have passed their final examination. They attend the Matron's course, which consists of lectures on " The General Details of Nursing," by Miss Lückes, every autumn. This course is succeeded by one on Surgical Nursing by Mr. Russell Howard, M.S., after Christmas, and this again by one in the third session on Medical Nursing by Dr. Lewis Smith. The lectures are given once a week from 8 to 9 p.m., and additional classes, with six in each class, are given by Sisters to probationers on the substance of the lectures. Two days a week the probationers gather for a study hour, lasting from 1.45 to 2.45 p.m. The final examination takes place in July, and is conducted by an outside examiner, Mr. Stephen Paget. Prizes are given. It is usually taken before the end of the first eighteen months, according to the time when the probationer began her training.

### The Last Six Months.

During this term of training the probationers have begun to be of value to the hospital. The aim now is to train them in the responsibilities of ward work, and they are known as " pro-staffs." The wards contain on an average twenty-eight beds, and of these a staff nurse has charge of fourteen and a pro-staff of the other fourteen, under the ward Sister. This post is held for three months on day duty and for three months on night duty, thus completing the formal training of the " London " nurses. It may be mentioned that during the two years each probationer has two spells of night duty, lasting in each case three months.

### Theatre Work.

The London Hospital possesses six general theatres—one ophthalmic, one aural, and two gynæcological, in addition to two general out-patients' theatres, with one aural and one ophthalmic. On the day we visited the training school twenty-one operations had taken place. Thus, the large number of nurses in training enjoy wide opportunities of witnessing surgical work and learning the best methods. It is the custom at the " London " for each Sister of a ward to take her own patients to the theatre, accompanied by a probationer or staff nurse. If probationers show promise they get the opportunity of accompanying cases and doing some simple duties in the theatre early in their training. In any case during the last six months they cannot fail to get this experience, and while on night duty as pro-staffs they witness every operation on patients in their ward which takes place at night.

### Extra Training.

Twelve pupil midwives can be afforded three months' training in the year, making forty-eight in all. Nurses are eligible to take this additional course *after* the first two years, and may either pay £21 for it, or add on an extra year's service, receiving their salary after, according to the position they are selected to fill. The private staff has now 110 nurses, who hold the C.M.B. certificate. A course of massage can also be taken, on condition of giving an extra year's service. Both these extra courses advance the nurses' prospects considerably, as will be shown.

### The Dual System.

After receiving her certificate of training the nurse, during the remaining two years of her term of service, serves as required, either as staff nurse in the wards or in some other department of the hospital, or as private nurse at outside cases. It is one of the features of the London Hospital that nurses in their third and fourth years are sent to cases direct from their wards, returning to ward work at the expiration of the engagement. All the nurses, whether in the hospital or at private work, sleep in the same Nurses' Home and dine together. During this final two years of her engagement a nurse nearly always finds valuable opportunities for enlarging her hospital experience. During the cases she takes outside the hospital she is in continual touch with the Matron, to whom she reports every week, and is thus launched into private work under very favourable conditions.

### The Hospital Staff.

After four years' service, nurses who devote themselves to institution work have many prospects of promotion awaiting them in their own hospital. As staff nurses they receive £24, rising annually £1 to £27. There are ninety-one of these staff nurses. The Sisters receive £30 in the first year, £35 in the second, and a maximum of £40 in the third. But after six years' service in the hospital they are entitled to an extra £5 a year, an annual bonus doubled at the end of twelve years' service. There are seventy-five Sisters and twelve holiday Sisters, and many good administrative posts are available, such as Matron's assistants, office assistants, etc., etc. A woman entering the hospital at the age of twenty-two might by the age of thirty be in receipt of £45 a year.

### Private Nursing.

The private staff consists of 272 nurses, including four who are always working in the massage department of the hospital. Throughout 1912 an average of about 214 nurses were employed at private cases. In all 2,503 cases were nursed, and 391 cases were refused. Nurses are sent for a period not exceeding eight weeks, and at the expiration of this time a higher fee is charged for their services should they still be required. Under no circumstances are they permitted to remain over three months at the same case. Nurses are entitled to four weeks' holiday in the year, and to a day off duty at the rate of one each fortnight, these days being usually taken together at the conclusion of a case.

Between their cases private nurses may be employed in the wards. Nurses on the private staff receive £30 in their third year, and rise by annual increments of £5 to £50. After six years' service an annual increment of £5 is given to each nurse, and after twelve years another £5 a year. In addition to these allowances all midwifery nurses who have paid the hospital £21 for their midwifery training are entitled to an extra £5 a year, whether they are doing any maternity work or not. The result of these annual bonuses may be stated as follows: A nurse entering the service of the hospital at the age of twenty-two, and taking the C.M.B. examination, would be entitled to £55 a year at the age of twenty-eight. Six years later, at the age of thirty-four, she would be entitled to £65 a year. And in another twelve years she could retire on full pay amounting to the average salary received during her last five years. For this pension the minimum age is forty-five, after eighteen years' service.

### The Nurses' Life.

The nurses' quarters at the London Hospital are finely planned, and afford ample opportunities for a well-ordered and harmonious community life. Separate bedrooms, charmingly fitted up, are available for each probationer and nurse. The common sitting-rooms are spacious and handsomely furnished, and the best possible use has been made of all the available space. The food is of good quality, well cooked and served, and the commissariat department is thoroughly efficient.

Probationers on day duty are allowed three hours off duty daily before 6 p.m., and one half-hour after the morning's work, in addition to meal times. When on night duty they have from 10.30 a.m. to 1 p.m. off duty. They are entitled to a whole day's holiday once a fortnight up to 10 p.m. without going into the wards at all. A late pass is granted occasionally on this day, and, if desired, breakfast may be had in bed. During each period of three months on night duty probationers may spend one night away from the hospital, returning by 1 p.m. the following day. On Sunday arrangements are made to enable all members of the nursing staff to attend one service at the hospital church, in addition to attendance at Holy Communion if desired. This does not mean, however, that attendance at church is compulsory. An extra hour off duty is allowed probationers on Sunday, in addition to time to attend church, and the hours on that day are somewhat different.

### The Aftermath.

The certificate of the London Hospital was first bestowed at the end of 1882, and from that day to the end of 1912 the total number of certificates of two years' training gained by London nurses is 2,234. What becomes of those nurses who pass away from their hospital at the end of their four years' agreement, or after long service, extending often to eighteen or twenty years? A glance at the supplementary register of the hospital will throw light on the prospects opened out to the nurses by their London Hospital training. They are scattered all over the world—in Australia, New Zealand, China, Chile, Uruguay, Canada, America, Perak, Africa, India, Newfoundland, France, Belgium, Denmark—holding for the most part important appointments. In Great Britain 169 London trained nurses are holding posts as matrons, nurse matrons, assistant matrons, sisters of general, cottage, and special hospitals and infirmaries; while many more are included in the Indian, Naval, and Military Nursing Services, and are holding posts as district nurses, as inspectors, health lecturers, and instructors in various departments of nursing. A certain number are at the head of Medical and Surgical Nursing Homes. The organised system through which nurses who have been trained at this hospital are kept in touch with it in after years entails an immense amount of additional labour on the Matron, and is of great public value. The admirable register of the London Hospital is worthy of wide imitation, and frequently proves a safeguard to the public.

